# Characteristics of the gut microbiome and metabolic profile in elderly patients with sarcopenia

**DOI:** 10.3389/fphar.2023.1279448

**Published:** 2023-11-03

**Authors:** Jing Zhou, Jiang Liu, Qinqing Lin, Linhui Shi, Zhigang Zeng, Lichang Guan, Yunzi Ma, Yingtong Zeng, Shilong Zhong, Lishu Xu

**Affiliations:** ^1^ Department of Geriatric Gastroenterology, Guangdong Provincial People’s Hospital (Guangdong Academy of Medical Sciences), Southern Medical University, Guangzhou, China; ^2^ Department of Pharmacy, Guangdong Provincial People’s Hospital (Guangdong Academy of Medical Sciences), Southern Medical University, Guangzhou, China; ^3^ College of Medicine, Shantou University, Shantou, China; ^4^ The Second School of Clinical Medicine, Southern Medical University, Guangzhou, China; ^5^ Department of Digestive Endoscopy Center, Guangdong Provincial People’s Hospital (Guangdong Academy of Medical Sciences), Southern Medical University, Guangzhou, China; ^6^ Guangdong Provincial Key Laboratory of Coronary Heart Disease Prevention, Guangdong Provincial People’s Hospital (Guangdong Academy of Medical Sciences), Southern Medical University, Guangzhou, China; ^7^ Laboratory of Phase I Clinical Trials, Center of Medical Research, Guangdong Provincial People’s Hospital (Guangdong Academy of Medical Sciences), Southern Medical University, Guangzhou, China; ^8^ Guangdong Provincial Institute of Geriatrics, Guangdong Provincial People’s Hospital (Guangdong Academy of Medical Sciences), Southern Medical University, Guangzhou, China

**Keywords:** sarcopenia, elderly patients, gut microbiota, metabolites, gut-muscle axis

## Abstract

**Introduction:** There is growing evidence of research indicating that the gut microbiota is involved in the development of sarcopenia. Nevertheless, there exists a notable deficiency in comprehension concerning the connection between irregularities in the intestinal microbiome and metabolic processes in older individuals suffering from sarcopenia.

**Methods:** To analyze fecal samples obtained from a cohort of 30 older patients diagnosed with sarcopenia as well as 30 older patients without sarcopenia, this study employed 16S rDNA sequencing and liquid chromatography-mass spectrometry (LC-MS)-based non-targeted metabolomics profiling techniques.

**Results:** As a result, we found that 29 genera and 172 metabolites were significantly altered in the sarcopenic patients. Among them, *Blautia*, *Lachnospiraceae_unclassified*, and *Subdoligranulum* were the bacteria with a potential diagnostic value for sarcopenia diagnosis. Correlation analysis between clinical indices and these gut bacteria suggested that the IL-6 level was negatively correlated with *Blautia*. Function prediction analysis demonstrated that 17 Kyoto Encyclopedia of Genes and Genomes (KEGG) pathways differ significantly between sarcopenic and non-sarcopenic patients. The primary classes of metabolites identified in the study included lipids and lipid-like molecules, organic acids and derivatives, and organoheterocyclic compounds. KEGG enrichment analysis showed that purine metabolism, arginine and proline metabolism, alanine, aspartate, and glutamate metabolism, butanoate metabolism, and histidine metabolism may contribute to the development of sarcopenia. The correlation study on gut microbiota and metabolites found that *Lachnospiraceae_unclassified* was positively associated with seven metabolites that were more abundant in the non-sarcopenia group and negatively correlated with three metabolites that were more abundant in the sarcopenia group. In addition, *Subdoligranulum* was positively correlated with seven metabolites that were lacking in sarcopenia and negatively correlated with two metabolites that were enriching in sarcopenia. Moreover, *Blautia* was positively associated with xanthosine.

**Discussion:** We conducted a study on the intestinal microbiota and metabolic profile of elderly individuals with sarcopenia, offering a comprehensive analysis of the overall ecosystem. Through this investigation, we were able to validate existing research on the gut–muscle axis and further investigate potential pathogenic processes and treatment options for sarcopenia.

## Introduction

Sarcopenia is a condition associated with aging that involves the pathological decline of muscle mass as well as significant decreases in both muscular strength and function. It commonly occurs in people >60 years of age and raises the economic burden of healthcare for the elderly according to the Asian Working Group for Sarcopenia criteria released in 2019 (AWGS 2019) (Chen et al., 2020). However, effective therapy for age-related sarcopenia is still limited due to a poor understanding of its pathogenesis to date. Given the global rise in the elderly demographic, it becomes imperative to investigate the holistic processes of aging and sarcopenia.

The importance of having a diverse gut microbiota is widely acknowledged as a crucial element necessary for preserving the normal functioning of the host’s body. Abnormal microbial composition can change key biological processes, including inflammation, metabolism, and nutrient absorption, thus adversely affecting muscle quality and function. Furthermore, imbalances in the gut microbiota due to age-related factors can disrupt the equilibrium of energy provision and nutritional support for muscles via the gut-muscle connection, leading to various disorders. There is emerging evidence that dysbiosis of the gut microbiota contributes to sarcopenia (Wang et al., 2022; Peng et al., 2023; Wang et al., 2023). Research has confirmed the presence of significant disparities in the diversity of the microbiota between older individuals afflicted with sarcopenia and those unaffected by sarcopenia (Lee et al., 2022). Further investigation shows that shifts in the makeup of gut bacteria, namely *Enterobacteriaceae*, *Bacteroides*, and *Prevotella*, have the potential to impact the functionality of skeletal muscles (Lustgarten, 2019). The alteration of the gut bacteria is related to a rise in pro-inflammatory cytokines (Picca et al., 2019). The correlation between muscles and the bacteria in the gut was confirmed by animal experiments (Lahiri et al., 2019).

Moreover, the corresponding syndrome of sarcopenia cannot be concluded to stem from gut microbiota in human senescence due to controversial findings about the linkage between gut microbiota and pathophysiological changes in the host. Therefore, to make this relationship clearer, it is necessary to verify the characteristic metabolites from the intestinal microenvironment of patients in addition to understanding the changes in microorganisms. Research has demonstrated that the gut microbiota possesses the ability to modulate the synthesis of diverse metabolites, including branched-chain amino acids (BCAA), short-chain fatty acids (SCFAs), protein synthesis, lipid metabolism, and carbohydrates (Liu C. et al., 2021), thereby influencing the metabolic processes of the host organism as well as the functionality of skeletal muscle tissue, and this observation supports the hypothesis of a gut–muscle axis (Zhao et al., 2021). The results of animal experiments also show that sarcopenia is closely related to the reduction in metabolic functionality of essential nutrients, including amino acids and folic acid (Siddharth et al., 2017). Moreover, the interaction between gut bacteria and dietary structure, especially protein intake, is of great significance for evaluating the impact of gut microbiota on muscle function (Ni Lochlainn et al., 2018; Prokopidis et al., 2020). However, there are few studies on sarcopenia caused by age, and the intestinal microflora has not been linked with fecal metabolic function or clinical indicators, providing additional evidence for the gut–muscle axis hypothesis.

Therefore, in order to further explore whether sarcopenia in the elderly is related to the changes of bacteria and metabolites in the intestine, our study adopted the latest diagnostic standard AWGS 2019 for sarcopenia and used 16S rDNA sequencing and non-targeted metabolomics of liquid chromatography-mass spectrometry (LC-MS) to perform a comprehensive analysis of the microbial community, fecal metabolites, and clinical indicators of sarcopenia in hospitalized elderly patients with sarcopenia. Our research aimed to reveal the shifts of microbiota and metabolites in the gut in elderly individuals with sarcopenia, as well as explore possible ties between gut bacteria, fecal metabolites, and clinical indicators.

## Materials and methods

The present prospective observational study was conducted after obtaining approval from the ethics committee of Guangdong Provincial People’s Hospital, China (approval no. GDREC20198345H(RI)). Before enrolling, all participants in the study gave their consent in writing after being informed.

### Study participants

This was a prospective observational study conducted in the wards of the Department of Geriatric Gastroenterology, Guangdong Provincial People’s Hospital in Guangzhou, China. We used the quota sampling method for sample informants. We recruited 30 older patients without sarcopenia and 30 older patients with sarcopenia from November 2021 to June 2022.

The participants underwent interviews conducted by staff members who had received specialized training. Every participant completed a comprehensive questionnaire that encompassed demographic factors, medical background, and details of prescribed medications. The inclusion criteria consisted of the following: 1) age >60 years; and 2) having a stable, healthy status. Exclusion criteria were as follows: 1) some diseases were not clearly diagnosed; 2) they received any antibiotic treatment within 1 month; or 3) they were unable to perform a body composition analysis.

According to the AWGS 2019, individuals suffering from sarcopenia were found to exhibit a primary diagnostic criterion characterized by a decrease in muscle mass. Additionally, they were observed to meet one of two secondary criteria, namely a decline in muscle strength or impaired physical capacity. In men, low muscle mass was characterized as an appendicular skeletal muscle index (ASMI) below 7.0 kg/m^2^, while in women, it was defined as below 5.7 kg/m^2^. The ASMI was determined through the division of the appendicular skeletal muscle mass by height squared. Body composition data were obtained by carrying out bioelectrical impedance analysis (TANITA MC-980MA; Tanitao Corp., Tokyo, Japan). The measurements were conducted by technologists with significant expertise in the field. Handgrip strength was evaluated by measuring muscle strength using the electronic grip dynamometer (CAMRY EH101; Zhongshan Camry Electronic Co., Ltd., Zhongshan, China). Each participant was directed to exert their maximum effort on two occasions, using both hands. The maximum value was utilized for subsequent studies. To classify grip strength as low, males must have less than 28 kg and women must have less than 18 kg. To evaluate the physical performance of the individuals, the traditional walking speed (m/s) during a 6-m walk test was utilized. Each participant was given instructions to perform two tests, and the test with the shorter duration was utilized to determine the presence of sarcopenia. The threshold for defining low gait speed was established as less than 1.0 m/s.

### Biochemical measurements

After fasting overnight, blood samples were collected and subsequently sent to the biochemistry laboratory at Guangdong Provincial People’s Hospital for clinical chemistry analysis. Various biochemical markers were assessed, such as albumin, pre-albumin, alanine aminotransferase (ALT), aspartate aminotransferase (AST), creatinine, glycated hemoglobin (HbA1c), triglycerides, uric acid, and 25-hydroxyvitamin D (25 [OH]D). In order to evaluate inflammation, the markers C-reactive protein (CRP) and interleukin-6 (IL-6) were examined.

### Fecal sample collection

Fecal samples were collected from all participants who were included in the study with the purpose of conducting 16S rDNA sequencing and metabolomics analysis. The specimens were promptly frozen and thereafter stored at a temperature of −80°C.

### DNA extraction and 16S rDNA sequencing

To obtain bacterial genomic DNA from fecal samples, the manufacturer’s guidelines were followed, utilizing hexadecyltrimethylammonium bromide for the extraction process. The effectiveness of the specialized reagent designed for extracting DNA from small amounts of samples has been proven in the context of DNA retrieval from various bacterial species. A blank medium consisting of water free from nuclear substances was utilized. The complete DNA sample was obtained by utilizing 50 μL of elution buffer and subsequently stored at a temperature of −80°C until it underwent measurement through a polymerase chain reaction (PCR).

In the research, the V3-V4 section of the 16S ribosomal RNA (rRNA) gene was amplified by utilizing primers 341F (5’-CCTACGGGNGGCWGCAG-3’) and 805R (5’-GACTACHVGGGTATCTAATCC-3’). The purpose of this amplification was to comprehensively analyze the bacterial makeup and quantity in patients with and without sarcopenia. The primers were modified at their 5′ends to include unique barcodes corresponding to each sample, along with universal sequencing primers. A reaction mixture with a total volume of 25 μL was utilized for conducting the PCR amplification. To adjust the volume, PCR-grade water was added to a mixture containing 25 ng of template DNA, 12.5 μL of PCR Premix, and 2.5 μL of each primer.

For the amplification of prokaryotic 16S fragments, the PCR utilized specific settings. These settings included an initial denaturation step at a temperature of 98°C for 30 s. Following this, a series of 32 cycles were performed. Each cycle consisted of denaturation at 98 °C for 10 s, annealing at 54°C for 30 s, and extension at 72°C for 45 s. Finally, a final extension step was conducted at 72°C for 10 min 2% agarose gel electrophoresis was used to confirm the PCR results. Ultrapure water was used as a negative control instead of a sample solution during the DNA extraction process to prevent false-positive PCR results. To purify the PCR products, AMPure XT beads (Beckman Coulter Genomics, Danvers, MA, United States) were utilized, and the quantification was conducted with Qubit (Invitrogen, Carlsbad, CA, United States). Preparation for sequencing was performed on the amplicon pools, and the Agilent 2100 bioanalyzer (Agilent Technologies, Santa Clara, CA, United States) and the Library Quantification Kit for Illumina (Kapa Biosciences, Woburn, MA, United States) were used to assess the size and quantity of the amplicon library. The Illumina NovaSeq platform was utilized for high-throughput sequencing.

### Analysis of 16S rDNA sequences

The sequencing of the samples was conducted on an Illumina NovaSeq platform following the manufacturer’s guidelines. The distinct barcode of each sample was utilized to identify paired-end reads, which were subsequently shortened by eliminating the barcode and primer sequence. Merging paired-end reads using fast length adjustment of short reads. The raw reads were filtered using fqtrim (version 0.94) to obtain clear, high-quality tags by applying specific conditions. The Vsearch (version 2.3.4) software was utilized to filter out chimeric sequences. After performing DADA2 deduplication, we acquired both a feature table and a feature sequence.

QIIME2 calculated alpha-diversity and beta-diversity using random normalization of identical sequences. As per the classifier provided by SILVA (release 138), the normalization of feature abundance was carried out based on the relative abundance values of the samples. The principal coordinate analysis (PCoA) employed the weighted UniFrac distance measure, whereas the *p*-value for ANOSIM was obtained through a permutation test. After aligning the BLAST sequence, the SILVA database was utilized to annotate the feature sequences of every representative sequence. Subsequently, we performed the Wilcoxon rank-sum test and employed linear discriminant analysis (LDA) effect size (LEfSe) analysis to ascertain the presence of distinct taxa. The measurement of areas under the receiver operating characteristic (ROC) curves (AUCs) of differential taxa was conducted using the R software (R Foundation for Statistical Computing, Vienna, Austria). The function of the differential taxa was predicted using phylogenetic investigation of communities by reconstruction of unobserved states 2 (PICRUSt2) and statistical analysis of metagenomic profiles (STAMP). Additional diagrams were implemented using R packages.

### Metabolites extraction and LC-MS analysis

We obtained 100 mg of feces by mixing it with 1 mL of 50% methanol that had been pre-cooled. The solution was agitated for 1 min and left at ambient temperature for 10 min. Following this, the extraction mixture was kept overnight at a temperature of −20°C. Following a 20-min period of centrifugation at a force of 4,000 times the force of gravity, the liquid above the sediment was cautiously moved to new 96-well plates. Pooled quality control (QC) samples were generated through the amalgamation of 10 μL from each extraction mixture.

The LC-MS system collected all samples in order. In this study, the Vanquish Flex UHPLC system (Thermo Fisher Scientific in Waltham, MA, United States) was utilized for all chromatographic separations. To carry out the reversed-phase separation, a column of ACQUITY UPLC T3 (100 mm × 2.1 mm, 1.8 µm; Waters, Milford, United States) was utilized. The column oven temperature was 35 °C. In the experiment, a flow rate of 0.4 mL/min was employed along with two solvents: solvent A, which consisted of water containing 0.1% formic acid, and solvent B, which was composed of acetonitrile with 0.1% formic acid. The conditions for gradient elution were as follows: from 0 to 0.5 min, 5% B; from 0.5 to 7 min, 5%–100% B; from 7 to 8 min, 100% B; from 8 to 8.1 min, 100%–5% B; from 8.1 to 10 min, 5%B.

The Q-Exactive (Thermo Fisher Scientific, Waltham, MA, United States) was used for both positive and negative ion analysis. To achieve an automatic gain control (AGC) target of 3–6, preceding spectra in the mass to charge ratio (*m/z*) range of 70–1050 were acquired with 70,000 resolutions. The injection time was limited to 100 ms. The data acquisition system was configured in DDA mode, utilizing a top-three configuration. To achieve an AGC target range of 1–5 with a maximum injection time of 80 milliseconds, fragment spectra were acquired at 17,500 resolutions. A QC sample (a pool of all samples) was acquired after every 10 samples to test the stability of the LC-MS system during data acquisition.

### Metabolomics analysis

The XCMS software was used to document the pre-treatment data of MS acquisition, which included peak selection, peak clustering, correction of retention time (RT), secondary peak clustering, and annotation of isotopes and adducts (Smith et al., 2006). The raw data files from LC-MS were converted to mzXML format and then processed using the XCMS, CAMERA, and metaX toolboxes, all of which were utilized in the R software. Integrating the RT and *m/z* data enabled the identification of every ion. A three-dimensional matrix was generated by recording the intensity of each peak. The matrix includes peak indices (RT–*m/z* pairs) that have been randomly assigned, as well as sample names (observations) and information about ion intensity (variables). The metabolites were identified by comparing the precise molecular weight information (*m/z*) of the samples with the corresponding data in the online Kyoto Encyclopedia of Genes and Genomes (KEGG) database and the Human Metabolome Database (HMDB). Metabolites were annotated if the difference in mass between the observed and database values was below 10 parts per million. Furthermore, the metabolite’s molecular formula was established and verified using isotopic distribution measurements. Additionally, we utilized an in-house-generated library of fragment spectra for metabolites to validate the identification of metabolites. The data at the highest level of intensity went through extra pre-processing with the metaX software. Any features that were detected in fewer than 50% of QC samples or fewer than 80% of biological samples were eliminated. To improve the overall data quality, the k-nearest neighbor algorithm was utilized to fill in the missing values for the remaining peaks. To identify anomalies and assess batch effects, the pre-processed dataset underwent principal component analysis (PCA). To mitigate signal intensity drift over time, a strong LOESS signal correction technique was employed to fit the QC data based on quality control, taking injection order into account. For all QC samples, metabolic feature relative standard deviations were calculated, and values over 30% were eliminated.

We employed Student's *t* tests to examine potential disparities in metabolite concentrations between two distinct groups. The discrimination of different variables between groups was achieved by conducting supervised partial least squares-discriminant analysis (PLS-DA) using the metaX software. The VIP value was computed, and a threshold value of 1.0 was employed to identify significant features. Differential metabolites were identified using *p*-values, VIP values, and fold change (FC) values. The KEGG enrichment analysis was conducted using MetaboAnalyst version 5.0, which can be accessed at http://www.metaboanalyst.ca. Additional diagrams were implemented using R packages.

### Statistical analysis

The differences between quantitative data were assessed using either a two-sided Student's *t* test or a Wilcoxon rank-sum test. Categorical variables were compared using Pearson’s chi-squared test, when applicable. Spearman’s correlation analysis was conducted to examine the associations between genera, metabolites, and clinical data. A significance level of *p* < 0.05 was deemed statistically significant, and the Benjamini–Hochberg (BH) method was employed to calculate a false discovery rate (FDR)-adjusted *p*-value, also known as a q-value. Statistical analyses were conducted using Microsoft Excel 2019 (Microsoft Corporation, Redmond, WA, United States) and R version 4.2.2, unless specified otherwise.

## Results

### General characteristics of participants

For the purposes of this investigation, a total of 60 participants were recruited, consisting of 30 individuals who did not exhibit symptoms of sarcopenia and 30 individuals who presented with sarcopenia. The two groups were paired based on several variables, including sex, smoking status, drinking status, body height, as well as levels of ALT, AST, creatinine, HbA1c, triglycerides, and uric acid. Nevertheless, given that sarcopenia is a condition associated with the aging process, it was seen that people belonging to the sarcopenia group had a noticeably greater average age when compared to those in the non-sarcopenia group. Furthermore, as compared to the group without sarcopenia, the group with sarcopenia had a significant reduction in anthropometric measures. These measures have served as the primary diagnostic markers for sarcopenia and include weight, body mass index (BMI), ASMI, handgrip strength, and performance on the 6-m walk test. In addition, the cohort identified as having sarcopenia had a significant decrease in the concentrations of albumin, pre-albumin, and 25(OH)D. This implies that there was a correlation between sarcopenia and inadequate nutrition. Furthermore, the group diagnosed with sarcopenia had a significant increase in systemic inflammatory markers, including CRP and IL-6. These findings were in line with previous research indicating a correlation between sarcopenia and inflammation (Tuttle et al., 2020). Table 1 displays the fundamental characteristics of the subjects.

**TABLE 1:** Demographic and clinical characteristics of participants.

	Non-sarcopenia (*n* = 30)	Sarcopenia (*n* = 30)	*p*-value
Age (years)	71.0 (65.2, 76.2)	91.0 (83.5, 92.0)	<0.001
Male, n (%)	21 (70)	18 (60)	0.417
Never smoke, n (%)	21 (70)	22 (73.3)	0.846
Never drink, n (%)	26 (86.7)	23 (76.7)	0.163
Height (cm)	164.0 ± 7.7	161.2 ± 7.7	0.157
Weight (kg)	66.3 ± 10.3	55.3 ± 9.4	<0.001
BMI (kg/m^2^)	24.5 ± 2.4	21.3 ± 3.5	<0.001
ASMI (kg/m^2^)	7.3 ± 0.9	5.5 ± 0.9	<0.001
Handgrip strength (kg)	28.2 (25.2, 35.1)	15.2 (0.0, 21.4)	<0.001
Six-meter walk (m/s)	1.2 (1.0, 1.3)	0.4 (0.0, 0.7)	<0.001
Albumin (g/L)	38.0 (37.0, 39.1)	34.8 (32.4, 36.4)	<0.001
Pre-albumin (g/L)	233.4 ± 36.7	196.2 ± 48.9	0.001
ALT (U/L)	17.5 (14.0, 23.8)	13.5 (11.0, 18.5)	0.058
AST (U/L)	21.1 ± 5.7	19.7 ± 6.7	0.364
Creatinine (μmol/L)	76.4 (66.2, 87.6)	87.7 (65.0, 98.8)	0.294
HbA1c (%)	6.2 (5.8, 6.6)	6.0 (5.7, 7.1)	0.988
Triglycerides (mmol/L)	1.0 (0.8, 1.7)	1.1 (0.8, 1.3)	0.631
Uric acid (μmol/L)	389.4 ± 84.5	346.2 ± 107.9	0.09
25(OH)D (ng/mL)	24.7 ± 7.0	18.0 ± 7.8	<0.001
CRP (mg/L)	0.8 (0.5, 1.5)	1.6 (0.7, 6.2)	0.022
IL-6 (pg/mL)	2.1 (1.5, 3.7)	5.5 (3.5, 10.5)	<0.001

BMI, body mass index; ASMI, appendicular skeletal muscle index; ALT, alanine aminotransferase; AST, aspartate aminotransferase; HbA1c, glycated haemoglobin; 25(OH)D, 25-hydroxyvitamin D; CRP, C-reactive protein; IL-6, Interleukin 6. Data are presented as the mean ± standard deviation (SD), median with interquartile range (IQR), or n (%). The *p* values are based on the two-sided Student’s *t* test for variables expressed as mean ± SD, the Wilcoxon rank-sum test for variables expressed as median (IQR), and Pearson’s chi-squared test for variables expressed as percentages.

### Gut microbiota changes in elderly patients with sarcopenia

We conducted high-throughput sequencing of the V3-V4 regions of the 16S rDNA to analyze and characterize the composition of the gut microbiota in elderly individuals, both with and without sarcopenia. From the fecal samples of 30 individuals without sarcopenia (consisting of 1,225,962 reads) and 30 individuals with sarcopenia (consisting of 1,279,770 reads), a total of 2,505,732 top-notch sequences were obtained.

**FIGURE 1:**
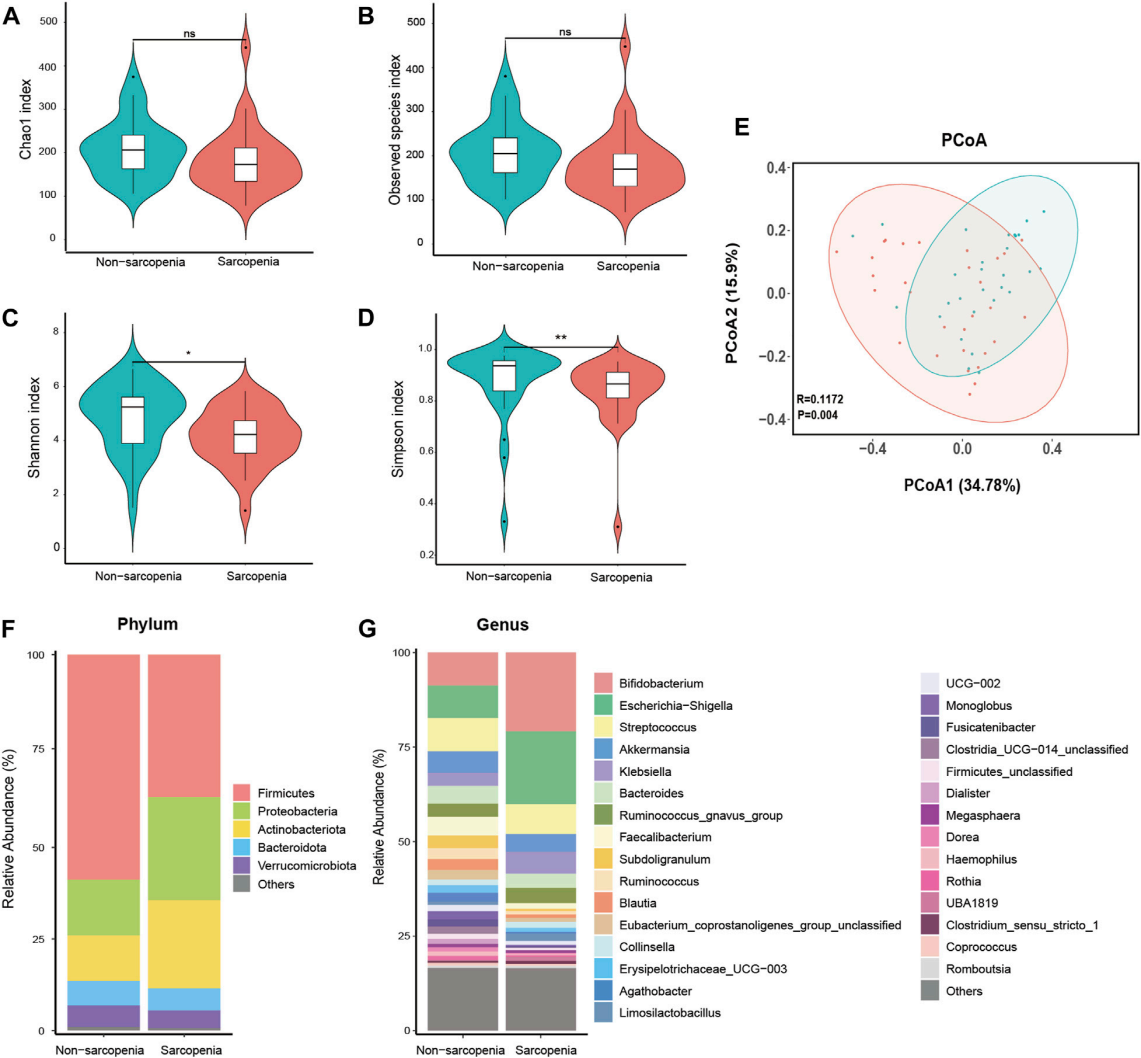
Comparative analysis of the diversity and relative abundance of species. Chao 1 index **(A)**, Observed species index **(B)**, Shannon index **(C)**, and Simpson index **(D)** were used to measure alpha-diversity. Violin plots show the median, quartiles, and min/max values. *p < 0.05, **p < 0.01, ***p < 0.001. ns, not significant. Principal coordinate analysis (PCoA) and analysis of similarities (ANOSIM) based on weighted UniFrac distance were used to measure beta-diversity **(E)**, *p* = 0.004, R = 0.1172. The distribution plot of relative abundance at the phylum level **(F)**, and the top 30 richest genera at the genus level **(G)**.

The DADA2 algorithm yielded a total of 3,981 feature sequences. The Chao1 and observed species indices, which are commonly used to assess the richness of the microbiota, did not show any statistically significant variation between the two experimental groups (Figures 1A, B). The Shannon and Simpson indices, which are indicators of microbial diversity, exhibited a statistically significant decrease in the sarcopenia group compared to the non-sarcopenia group (Figures 1C, D). The microbial community structure between the two groups showed substantial differences, as observed by principal coordinate analysis and ANOSIM using weighted UniFrac distance metrics (*p* = 0.004, R = 0.1172, Figure 1E).

The dysbiosis of gut microbial composition in patients with sarcopenia was identified using a comparative examination of relative abundance at the phylum and genus levels. The Wilcoxon rank-sum test was employed to ascertain the taxa that exhibited statistically significant alterations. At the phylum level, the sarcopenia group exhibited a statistically significant decrease in the abundance of *Firmicutes* and a corresponding increase in the abundance of *Proteobacteria* (Figure 1F). Among the entire count of 616 genera, a specific group of 47 genera displayed fluctuating levels of abundance at the genus level. Among the variously abundant genera, the sarcopenia group showed a decrease in abundance for 26 genera and an increase in abundance for 21 genera (Supplementary Table S1). After *p*-value adjustment, *Blautia* and *Lachnospiraceae_unclassified* remained significantly decreased in the sarcopenia group. Thirteen genera exhibited notable variations in abundance within the top 30 most affluent genera (Figure 1G; Supplementary Table S2).

**FIGURE 2:**
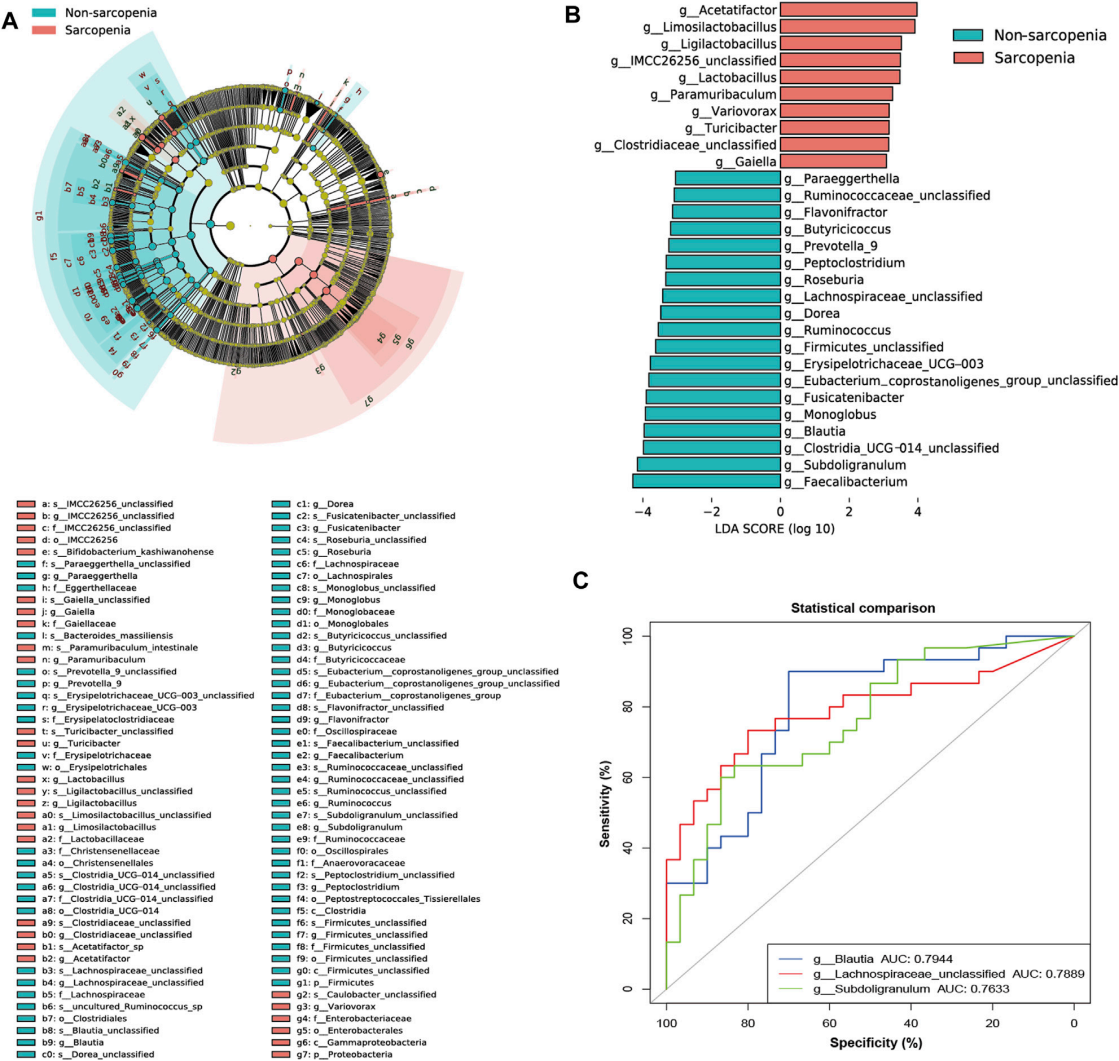
The specific altered taxa identified by linear discriminant analysis (LDA) effect size (LEfSe) analysis (LDA >3, *p* < 0.05) and the areas under the receiver operating characteristic (ROC) curve (AUC) of the top three AUC values of the differential genera in LEfSe. The phylogenetic tree in a cladogram of the specific differential taxa **(A)**. The histogram of 10 genera enriched in the sarcopenia group and 19 genera enriched in the non-sarcopenia group **(B)**. ROC curves and AUC values of *Blautia*, *Lachnospiraceae_unclassified*, and *Subdoligranulum* **(C)**.

To more precisely determine the predominant microbes linked with sarcopenia, we employed LEfSe to compare the microbial makeup of the two groups. In total, 94 genera were substantially recognized as notably discriminative (LDA >3, *p* < 0.05, Figure 2A). At the genus level, 29 distinct genera were recognized. Specifically, 10 genera were more abundant and 19 genera were less abundant in the sarcopenia group. The patients with sarcopenia exhibited a higher abundance of *Acetatifactor* and *Limosilactobacillus* genera, whereas the patients without sarcopenia showed a higher abundance of *Faecalibacterium*, *Subdoligranulum*, *Clostridia_UCG_014_unclassified*, and *Blautia* genera (Figure 2B; Supplementary Table S3).

The apparent difference in gut microbiome makeup among older individuals with sarcopenia and those without sarcopenia presents the fascinating potential of using gut microbiota as potential indicators for the identification and anticipation of sarcopenia. To achieve this objective, we have utilized the relative abundances of the aforementioned potential taxa as predictors of sarcopenia. ROC curve analysis was used to evaluate the AUC in order to compare the differences in gut microbiome composition between individuals with sarcopenia and those without sarcopenia. As a result, *Blautia*, *Lachnospiraceae_unclassified*, and *Subdoligranulum* were ranked high, as seen by their respective AUC values of 0.7944, 0.7889, and 0.7633 (Figure 2C; Supplementary Table S3).

**FIGURE 3:**
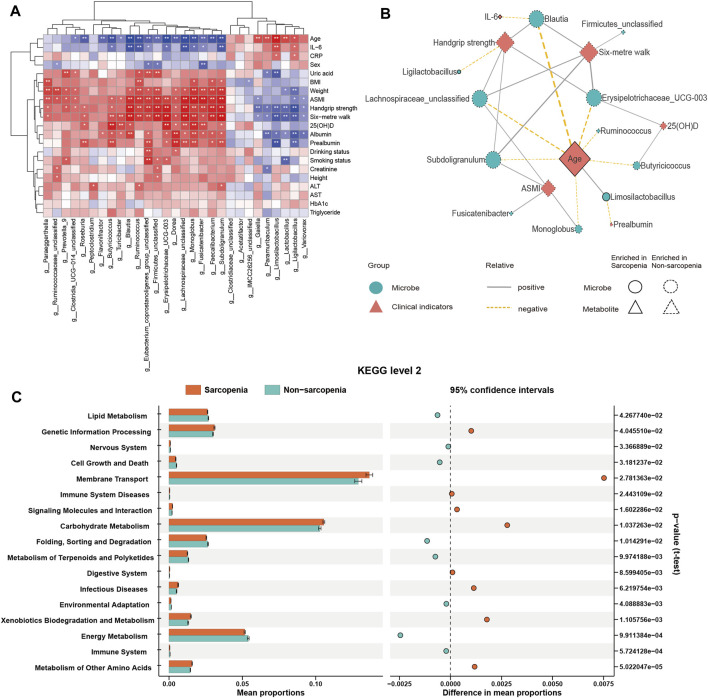
Correlations between distinct genera and clinical parameters and function prediction of distinct genera. Spearman’s correlation analysis of distinct genera and clinical parameters **(A)**, *p < 0.05, **p < 0.01, ***p < 0.001. Co-occurrence network graph of the main distinct genera and clinical parameters **(B)**, |r| > 0.45, *p* < 0.05. Genera are labeled with circles, while clinical parameters are labeled with diamonds. The nodes with solid or dashed boundaries were enriched in patients with or without sarcopenia, respectively. Positive correlations are shown by gray and solid connecting lines, whereas negative correlations are represented by yellow and dashed connecting lines, and thicker lines indicate higher correlation values. The Kyoto Encyclopedia of Genes and Genomes (KEGG) pathways with significant differences in function prediction using Statistical Analysis of Metagenomic Profiles (STAMP) software **(C)**.

To investigate the connection between the variably abundant genera and clinical parameters, a Spearman’s correlation analysis was performed. The findings were then presented using a heatmap (Figure 3A; Supplementary Table S3). The primary associations (|r| > 0.45, *p* < 0.05) were additionally visualized in a network graph depicting co-occurrence (Figure 3B). It was shown that age, a possible confounding factor in the experiment, had significant impacts on nearly all genera that were enriched in sarcopenia, such as *Blautia*, *Erysipelotrichaceae_UCG-003*, *Lachnospiraceae_unclassified*, *Limosilactobacillus*, *Ruminococcus*, *Subdoligranulum*, *Butyricicoccus*, and *Monoglobus*. A negative correlation was seen between the pre-albumin level and *Limosilactobacillus*, while a negative correlation was also found between the IL-6 level and *Blautia*. Moreover, a positive relationship was observed between the 25(OH)D level and the abundance of *Erysipelotrichaceae_UCG-003* and *Butyricicoccus*.

Predictions concerning the possible functional consequences of the observed changes in the microbiome of sarcopenic individuals were made using the PICRUSt2 analysis approach. According to the STAMP analysis, 17 KEGG pathways exhibited statistically significant alterations when comparing patients with sarcopenia to those without this condition (Figure 3C; Supplementary Table S5). The findings showed a notable enrichment in pathways associated with the metabolism of other amino acids and xenobiotics biodegradation and metabolism in sarcopenic patients. Conversely, pathways associated with the immune system and energy metabolism were found to be deficient in these individuals.

### Gut metabolites changes in elderly patients with sarcopenia

Considering the possible influence of the gut microbiome on metabolic pathways and the immune system via the generation of metabolites (Krautkramer et al., 2021), a non-targeted metabolomics profiling using LC-MS was conducted on the fecal samples collected from all individuals involved in the study. In the end, a grand total of 3,028 characteristics were verified as genuine by utilizing the MS2 fragment spectrum, with 2,592 characteristics being effectively measured.

**FIGURE 4:**
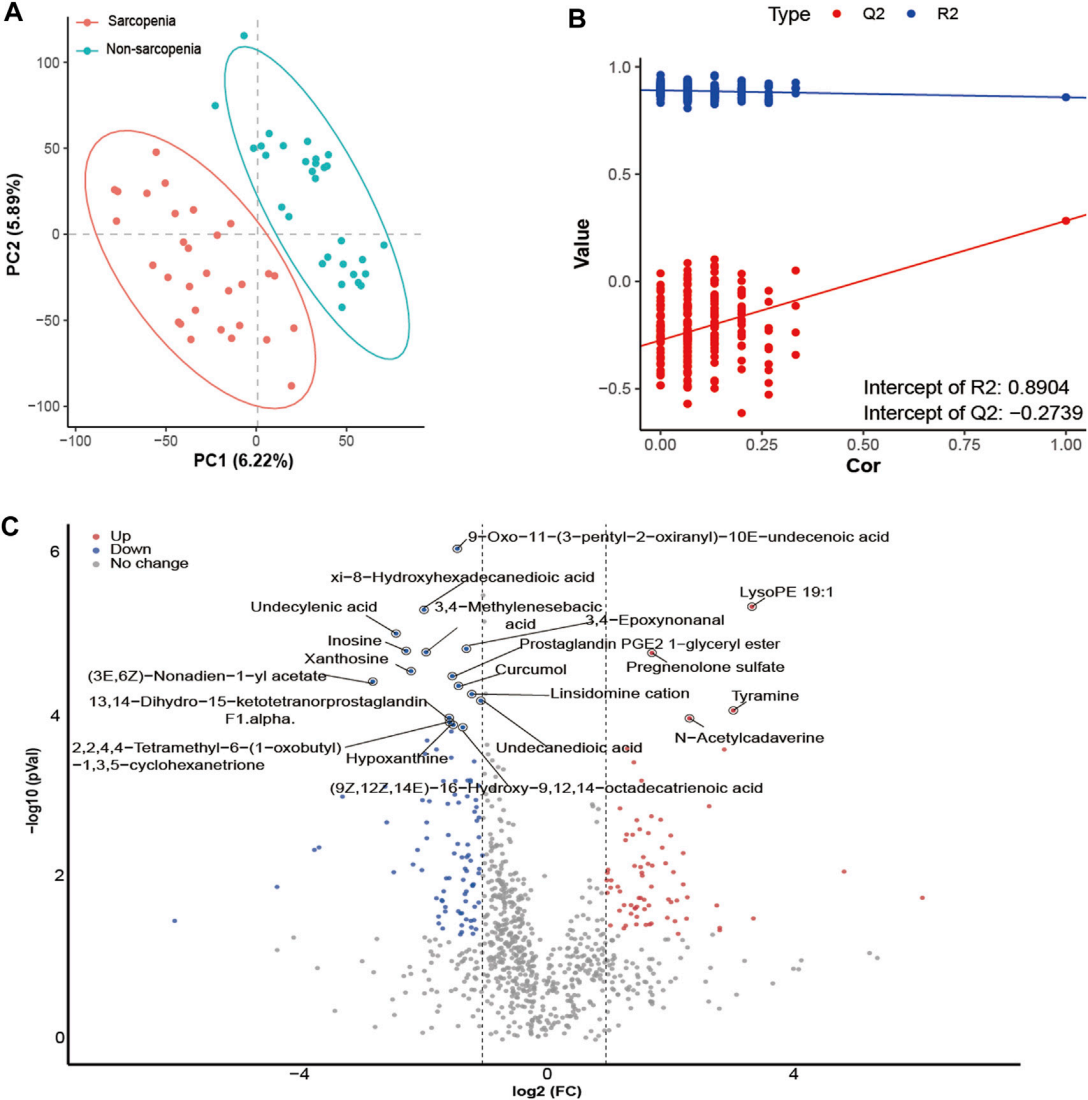
Alterations in fecal metabolites in sarcopenic patients. The partial least-squares-discriminant analysis (PLS-DA) score plot of the first two principal components **(A)**. Validation model through 200 permutation tests **(B)**. Volcano plot of differentially abundant metabolites between the sarcopenia group and the non-sarcopenia group **(C)**. Red spots represent metabolites enriched in patients with sarcopenia, blue spots represent metabolites enriched in patients without sarcopenia, and gray spots indicate that there were no significant differences between the two groups. Twenty metabolites remained significantly different after *p*-value adjustments were labeled.

The differential abundance of metabolites was determined with the application of multivariate analysis. The PLS-DA model successfully discriminated between patients with and without sarcopenia based on their distinct metabolic profiles (Figure 4A). Based on the permutation test results, it can be inferred that the PLS-DA model did not demonstrate overfitting (Intercept of Q2 = −0.2739, Figure 4B). Consequently, the comparative analysis between individuals diagnosed with sarcopenia and those without this condition led to the successful discovery of 172 metabolites with varying abundance (VIP >1, FC > 2 or <0.5, *p* < 0.05, Figure 4C; Supplementary Table S6). The primary classes of metabolites identified in the study included lipids and lipid-like molecules, organic acids and derivatives, and organoheterocyclic compounds. After *p*-value adjustment, 20 metabolites remained significantly different, and the sarcopenia group exhibited significantly high levels of lysoPE 19:1, pregnenolone sulfate, tyramine, and n-acetylcadaverine, while metabolites enriched in the non-sarcopenia group included 9-oxo-11-(3-pentyl-2-oxiranyl)-10E-undecenoic acid, xi-8-hydroxyhexadecanedioic acid, undecylenic acid, 3,4-epoxynonanal, inosine, 3,4-methylenesebacic acid, xanthosine, prostaglandin PGE2 1-glyceryl ester, (3E,6Z)-nonadien-1-yl acetate, curcumol, linsidomine cation, undecanedioic acid, 13,14-dihydro-15-ketotetranorprostaglandin F1 alpha, 2,2,4,4-tetramethyl-6-(1-oxobutyl)-1,3,5-cyclohexanetrione, hypoxanthine, and (9Z,12Z,14E)-16-hydroxy-9,12,14-octadecatrienoic acid (Figure 4C). Notably, 10 of these 20 metabolites belong to the lipid and lipid-like molecule class.

Spearman’s correlation analysis was employed to evaluate the potential influence of clinical factors on differentially abundant metabolites and demonstrate their association (Supplementary Table S7). Consequently, there were notable correlations seen between age and the levels of inosine, xanthosine, and pregnenolone sulfate. Moreover, a notable association was found between the 6-m walk assessment and both undecylenic acid and xanthosine. Additionally, handgrip strength exhibited a positive correlation with xanthosine, whereas albumin levels showed a positive correlation with undecylenic acid.

**FIGURE 5:**
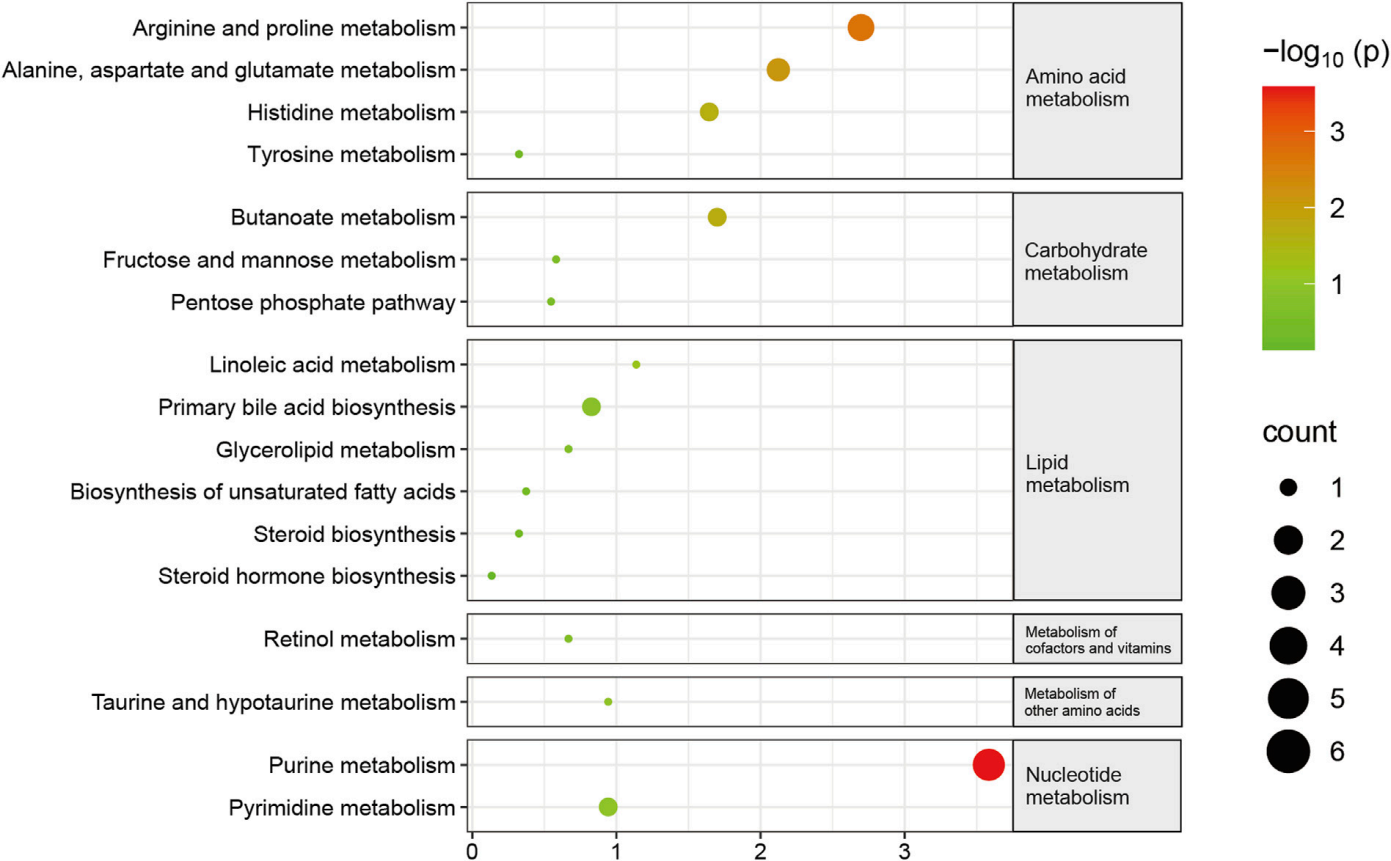
Pathway changes in the Kyoto Encyclopedia of Genes and Genomes (KEGG) in sarcopenic patients. The KEGG pathway enrichment scatterplot demonstrates changes in the gut metabolic processes of sarcopenic patients.

Subsequently, KEGG pathway enrichment analysis was conducted on metabolites that exhibited differential abundance. Consequently, a total of seventeen pathways exhibited considerable enrichment. The primary pathways associated with sarcopenia include purine metabolism, arginine and proline metabolism, alanine, aspartate, and glutamate metabolism, butanoate metabolism, and histidine metabolism (Figure 5, Supplementary Table S8). A total of fourteen metabolites exhibiting variable abundance were found to be implicated in the five main pathways. Patients diagnosed with sarcopenia displayed elevated levels of eight metabolites, including gamma-aminobutyric acid, 2-hydroxyglutarate, 1-pyrroline-5-carboxylic acid, n-acetyl-l-aspartic acid, n-acetylputrescine, methylimidazoleacetic acid, agmatine, and imidazole-4-acetaldehyde. Besides, it was observed that the levels of six metabolites, including hypoxanthine, guanine, inosine, guanosine, xanthosine, and xanthine, were found to be reduced in patients with sarcopenia.

### Multiomics analysis revealed the interactions between sarcopenia-related microbiota and metabolites

**FIGURE 6:**
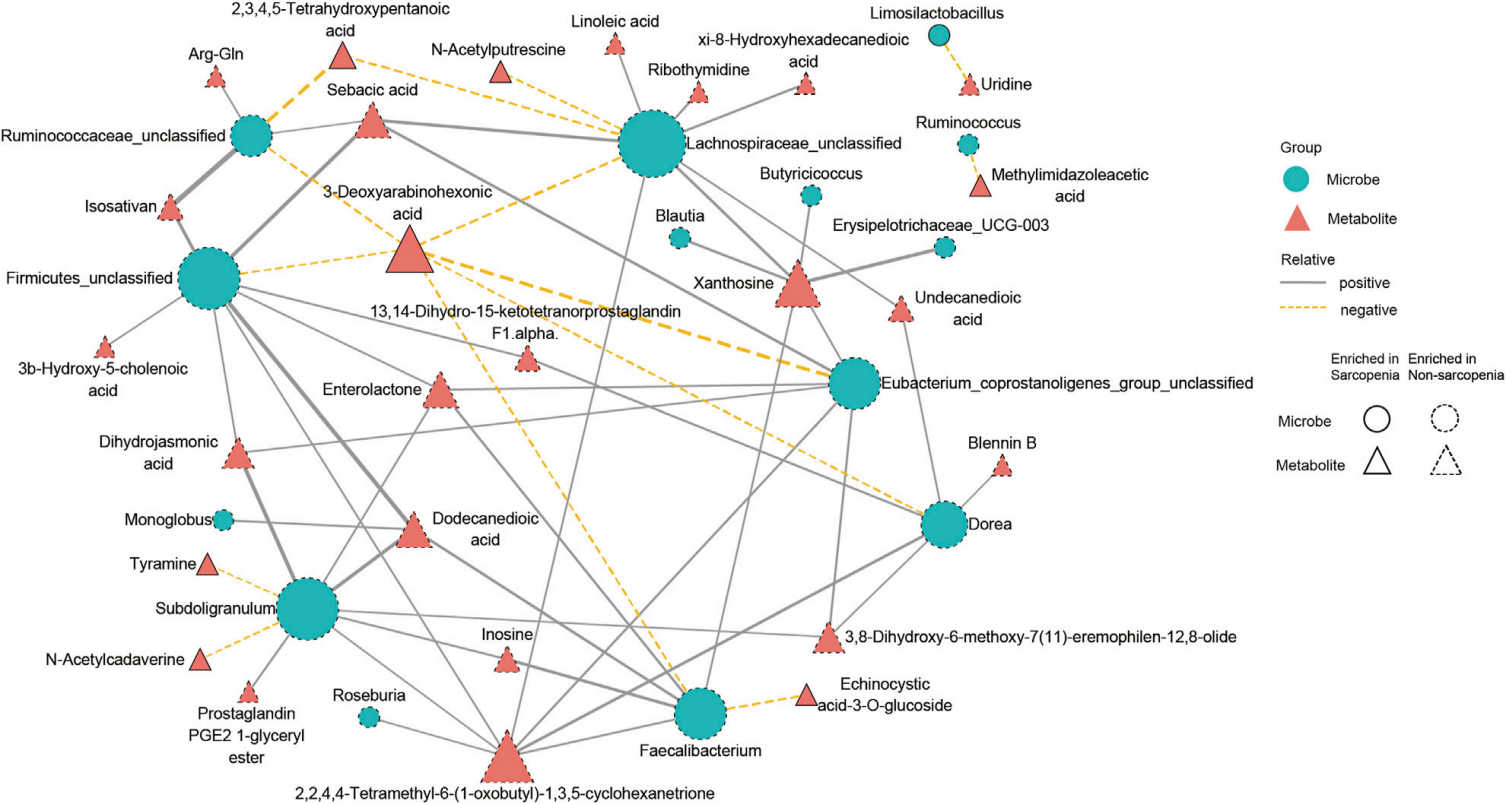
A multiomics method showed that sarcopenic patients had interactions between microbiota and metabolites. The main links between bacteria and metabolites are shown in the co-occurrence network graph (|r| > 0.55, *p* < 0.05). Microbes are labeled with circles, while metabolites are labeled with triangles. The nodes with solid or dashed boundaries were enriched in patients with or without sarcopenia, respectively. Positive correlations are shown by gray and solid connecting lines, whereas negative correlations are represented by yellow and dashed connecting lines, and thicker lines indicate higher correlation values.

To conduct a more comprehensive examination of the relationships between microbiota and metabolites associated with sarcopenia, we carried out an analysis to evaluate the associations between 29 distinctively abundant genera and 172 differentially abundant metabolites (Spearman’s correlation analysis, Supplementary Table S9). Afterwards, a network graph of co-occurrences was created to visually represent the main interactions (|r| > 0.55, *p* < 0.05, Figure 6). The graphical representation suggests that *Lachnospiraceae_unclassified*, *Firmicutes_unclassified*, *Subdoligranulum*, *Faecalibacterium*, *Eubacterium_coprostanoligenes_group_unclassified*, *Dorea*, and *Ruminococcaceae_unclassified* appear to be the central genera, as they exhibit correlations with several metabolites. Remarkably, *Lachnospiraceae_unclassified* was positively correlated with seven metabolites (sebacic acid, xanthosine, xi-8-hydroxyhexadecanedioic acid, ribothymidine, undecanedioic acid, 2,2,4,4-tetramethyl-6-(1-oxobutyl)-1,3,5-cyclohexanetrione, and linoleic acid) enriched in the non-sarcopenia group and negatively correlated with three sarcopenia-enriched metabolites (3-deoxyarabinohexonic acid, 2,3,4,5-tetrahydroxypentanoic acid, and N-acetylputrescine). In addition, *Subdoligranulum* was positively correlated with seven metabolites (dihydrojasmonic acid, dodecanedioic acid, inosine, prostaglandin PGE2 1-glyceryl ester, enterolactone, 3,8-dihydroxy-6-methoxy-7(11)-eremophilen-12,8-olide, and 2,2,4,4-tetramethyl-6-(1-oxobutyl)-1,3,5-cyclohexanetrione) that were lacking in sarcopenia and negatively correlated with two metabolites (N-acetylcadaverine and tyramine) that were enriching in sarcopenia. Finally, *Blautia* was positively associated with xanthosine.

## Discussion

In a study observing elderly patients who were hospitalized, we examined the characteristics of the gut microbiome and metabolome in individuals with sarcopenia. Consistent with previous studies on sarcopenia in elderly individuals, our research noted that the fecal microbiota and metabolites of sarcopenic patients showed significant differences compared to those of non-sarcopenic patients. As demonstrated in the results, compared to the non-sarcopenia group, the sarcopenia group had lower gut microbial diversity. *Blautia*, *Lachnospiraceae,* and *Subdoligranulum* decreased in the sarcopenia group. Lipids and lipid-like molecules, organic acids and derivatives, and organoheterocyclic compounds also showed significance in sarcopenia pathogenesis. Pathways related to purine metabolism, arginine and proline metabolism, alanine aspartate and glutamate metabolism, butanoate metabolism, and histidine metabolism were found in differentially abundant metabolites. Except for dysbiosis, we also found strong associations between sarcopenia and increasing inflammation, malnutrition, and a reduced vitamin D level.

The sarcopenia group showed less alpha- and beta-diversity, meaning that sarcopenia is related to a decline in microbiota diversity. Actually, many studies have proven that the aging process is followed by dynamic microbiota changes, and the microbiota characteristics may explain the aging process and may become a therapeutic target (Vaiserman et al., 2017). As sarcopenia is an age-related disease, the specific bacterial differences excited our interest. Based on the ROC result, *Blautia*, *Lachnospiraceae*, and *Subdoligranulum* were the bacteria with the highest ROC areas, which were all downregulated in the sarcopenia group. These might indicate the potential diagnostic value of these three bacteria for sarcopenia diagnosis. *Blautia* is an important genus of the gut microbiota community and is regarded as a probiotic owing to its ability to alleviate inflammation and metabolic diseases (Liu X. et al., 2021). *Blautia* abundance negatively correlates with age (Odamaki et al., 2016) and nutritional status (Fluitman et al., 2022). Our research revealed a decline in *Blautia* abundance in the sarcopenia group, which might lead to chronic inflammation and metabolic or nutrition dysregulation in sarcopenic patients. The *Lachnospiraceae* family is also important for human health, and there are previous studies revealing a positive association between *Lachnospiraceae* and muscle mass (Liu C. et al., 2021). In colorectal carcinoma cases, *Lachnospiraceae* promoted the immune surveillance ability of CD8^+^ T-cells, thereby inhibiting cancer progression (Zhang et al., 2023). The positive effect of *Lachnospiraceae* on aging muscle might be related to its immune-regulation function. *Subdoligranulum* is a bacterium that remains controversial. In prior research, *Subdoligranulum* showed a strong association with abnormal inflammation and immune status (Chriswell et al., 2022). Our results showed that *Blautia*, *Lachnospiraceae*, and *Subdoliglanum* are related to inflammation. In our clinical indicators, we also found a positive correlation between the inflammation-related index IL-6 and the upregulation of bacteria and metabolites in the sarcopenia group. Previous studies have shown that pro-inflammatory biomarkers, including IL-6, IL-10, IL-8, IL-15 (Pan et al., 2021), CRP, TNF-α, and GDF-15 (Tuttle et al., 2020), are associated with skeletal muscle decline and sarcopenia. There is currently convincing evidence that inflammation is related to aging and sarcopenia (Larsson et al., 2019; López-Otín et al., 2023). Abnormal microbial composition may be an important cause of inflammation in patients with sarcopenia. In addition, *Subdoligranulum* has been recognized as a butyrate producer and is positively associated with muscle mass due to butyrate and its metabolites (Han et al., 2022). Okamoto et al. carried out experiments on SCFAs generated by gut microbiota (Okamoto et al., 2019); specifically, they used antibiotics to clear the gut microbiota of mice and found that the exercise endurance of mice was significantly reduced. However, the exercise endurance of mice could be restored by introducing SCFAs into their systems. This study shows that SCFAs produced in the intestine may be important for muscle function. Consistent with previous findings, in our study, the microbiota (*Eubacterium*, *Roseburia*, *Butyricicoccus*, *Anaerostipes*, and *Achnospiraceae*) producing SCFAs combined with sarcopenia were significantly reduced. Fecal metabolites are also enriched in the butyric acid metabolic pathway. This phenomenon shows that changes in gut microbiota not only regulate skeletal muscle quality through inflammatory cytokines but also regulate the gut–muscle axis by changing the SCFAs.

In the study on metabolites, we found that several metabolites showed significantly different enrichment between the sarcopenia and non-sarcopenia groups, including lipids and lipid-like molecules, organic acids and derivatives, and organoheterocyclic compounds. In the lipid and lipid-like molecule class, we found that the levels of glycerophosphates (lysoPE 19:1, lysoPE 17:1, and lysoPC 20:1) in sarcopenic patients were significantly increased. Lipids play an important role in transmembrane transport, energy metabolism, signal transduction, and the occurrence and development of many diseases (Bargui et al., 2021). There was a consistent observation of glycerophosphate disruption, specifically lysoPE 19:1, in the gut microbiota of patients diagnosed with rheumatoid arthritis (Yu et al., 2021). Our results revealed that many metabolites related to phospholipid metabolism have changed significantly, which may reflect phospholipid changes in skeletal muscle cell membranes. Sarcopenia may affect the metabolism of glycerol and phospholipids.

In addition, in the lipid and lipid-like molecule grouping, we also found that the fecal cholesterol concentration of sarcopenic patients was increased. Consistent with the serum results, some studies have found that the cholesterol level of people with sarcopenia increases, indicating that lipid metabolism may affect the onset and progression of sarcopenia (Gong et al., 2022). Lipid accumulation in muscular tissue was associated with inflammation and finally muscle atrophy (Li et al., 2022). The organic acid and derivative group ranked second in terms of the content of metabolites in sarcopenia, among which polyamine metabolites (N-acetylecadavine and N-acetyleputrescine) were significantly upregulated. Polyamines (cadaverine, spermine, and putrescine) are important components of many physiological activities of the human body (Handa et al., 2018). Studies have found that polyamine levels change in myasthenia gravis (Szathmáry et al., 1994). The organoheterocyclic compound group ranked third in the content of metabolites in sarcopenia, among which the level of hypoxanthine decreased significantly. Also, KEGG analysis indicated metabolites’ remarkable enrichment of purine metabolism. The purine family is mainly related to ATP metabolism, uric acid synthesis, and inflammation regulation (Linden et al., 2019). Xanthine and hypoxanthine are key intermediates in purine metabolism in the human body, which are catalyzed by xanthate oxidoreductase to produce uric acid. Some studies have shown that the serum urate level in sarcopenic patients is reduced (Molino-Lova et al., 2017; Liu et al., 2022). In our findings, although the blood uric acid level was not statistically significant between the two groups, it was evident that the average blood uric acid value was lower in patients with sarcopenia compared to those without sarcopenia. It means that the trend in the change in blood uric acid level aligns with previous findings reported in the literature.

Our clinical index detection showed that nutritional indices (such as albumin and pre-albumin) and vitamin D levels correlate with gut microbiota. Studies have shown that gut microbiota may reduce nutritional bioavailability, albumin, leucine, and metabolites. Undernutrition and vitamin D deficiency are recognized as underlying causes of aging muscle loss and even sarcopenia (Chen et al., 2022). For nutrition status, a previous study suggested that a lower abundance of *Blautia* was associated with undernutrition (Fluitman et al., 2022), which supported our result showing a positive relationship between *Blautia* abundance and the muscle index. Moreover, the metabolic pathways of arginine and proline metabolism and alanine aspartate and glutamate metabolism in fecal metabolites have also changed, which also proves that microorganisms affect the bioavailability of nutrients. Sarcopenia onset is associated with a lack of vitamin D, which is crucial for the proper functioning of the musculoskeletal system. According to a population study conducted in Japan, it was discovered that a decline in vitamin D levels is linked to reduced muscle strength. Additionally, the muscle strength in gene-knockout mice is affected by impaired signals related to vitamin D (Mizuno et al., 2022). Experiments on aged mice proved that vitamin D deficiency induced the sarcopenia phenotype, probably by downregulating the Notch signaling pathway (Domingues-Faria et al., 2014). Therefore, our findings indicate that the gut microbiota may affect sarcopenia through nutritional status and vitamin D concentration.

Some limitations of this research should be noted. First, as a cross-sectional study, longitudinal data were missing, and causality could not be proved. A longitudinal study including more elderly participants with variant characteristics is needed to decipher the time course of sarcopenia progression and the dynamic alteration of skeletal muscles. Second, our data only reveal the gut microbiome and metabolome features of patients in a single hospital. Further multi-center and multi-area research should be carried out for a better explanation of general sarcopenia in elderly people. Third, considering that this study strictly selected hospitalized participants based on criteria, albeit with a balanced baseline, the data might not be representative of home-dwelling elderly people, including those receiving nursing care at home. More data is needed to generalize the results to more populations. Fourth, we only analyzed the fecal metabolites. Current evidence indicates that circulating metabolites will also affect the skeletal muscles (He et al., 2023).

Taken together, this is a prospective observational study to elaborate on the microbiome and metabolome characteristics of hospitalized elderly sarcopenic patients. The result indicated that patients with sarcopenia have distinguishable microbiome, metabolome, and clinical characteristics compared to those without sarcopenia. This study verified previous research on the gut–muscle axis and explored potential pathological mechanisms and therapeutic targets of sarcopenia. Longitudinal research, animal-targeted experiments, and clinical interventional trials are needed for further mechanism exploration.

## Data Availability

The datasets generated for this study can be found in the SRA of NCBI: https://www.ncbi.nlm.nih.gov/sra/PRJNA1005560, and MetaboLights: https://www.ebi.ac.uk/metabolights/MTBLS8332.
